# Involvement of chemokine receptor CXCR3 in the defense mechanism against *Neospora caninum* infection in C57BL/6 mice

**DOI:** 10.3389/fmicb.2022.1045106

**Published:** 2023-01-10

**Authors:** Hanan H. Abdelbaky, Shuichiro Mitsuhashi, Kenichi Watanabe, Nanako Ushio, Miku Miyakawa, Hidefumi Furuoka, Yoshifumi Nishikawa

**Affiliations:** ^1^National Research Center for Protozoan Diseases, Obihiro University of Agriculture and Veterinary Medicine, Obihiro, Hokkaido, Japan; ^2^Division of Pathobiological Science, Department of Basic Veterinary Medicine, Obihiro University of Agriculture and Veterinary Medicine, Obihiro, Hokkaido, Japan; ^3^Laboratory of Veterinary Pathology, Department of Veterinary Medicine, Obihiro University of Agriculture and Veterinary Medicine, Obihiro, Hokkaido, Japan

**Keywords:** *Neospora caninum*, neosporosis, CXCR3, CXCR3KO, IL-6, IFN-γ, regulatory T-cells

## Abstract

C-X-C motif chemokine receptor 3 (CXCR3) is an important receptor controlling the migration of leukocytes, although there is no report regarding its role in *Neospora caninum* infection. Herein, we investigated the relevance of CXCR3 in the resistance mechanism to *N. caninum* infection in mice. Wild-type (WT) C57BL/6 mice and CXCR3-knockout (CXCR3KO) mice were used in all experiments. WT mice displayed a high survival rate (100%), while 80% of CXCR3KO mice succumbed to *N. caninum* infection within 50 days. Compared with WT mice, CXCR3KO mice exhibited significantly lower body weights and higher clinical scores at the subacute stage of infection. Flow cytometric analysis revealed CXCR3KO mice as having significantly increased proportions and numbers of CD11c-positive cells compared with WT mice at 5 days post infection (dpi). However, levels of interleukin-6 and interferon-γ in serum and ascites were similar in all groups at 5 dpi. Furthermore, no differences in parasite load were detected in brain, spleen, lungs or liver tissue of CXCR3KO and WT mice at 5 and 21 dpi. mRNA analysis of brain tissue collected from infected mice at 30 dpi revealed no changes in expression levels of inflammatory response genes. Nevertheless, the brain tissue of infected CXCR3KO mice displayed significant necrosis and microglial activation compared with that of WT mice at 21 dpi. Interestingly, the brain tissue of CXCR3KO mice displayed significantly lower numbers of FoxP3^+^ cells compared with the brain tissue of WT mice at 30 dpi. Accordingly, our study suggests that the lack of active regulatory T cells in brain tissue of infected CXCR3KO mice is the main cause of these mice having severe necrosis and lower survival compared with WT mice. Thus, CXCR3^+^ regulatory T cells may play a crucial role in control of neosporosis.

## Introduction

*Neospora caninum* is an obligate intracellular protozoan parasite. Canines are the definitive host, while the *Bovidae* family is the main intermediate host. *Neospora caninum* infection is characterized by neurological disorders in dogs and stormy abortion in cattle. Currently, no effective vaccine or commercial treatments are available for bovine neosporosis. Identifying *N. caninum* molecules responsible for pathogenesis or participating in the host defense mechanism against infection is essential for establishing new control strategies (Dubey et al., 2017).

The chemokines CXCL9, CXCL10, and CXCL11 are related molecules within the non-ELR (glutamic acid-leucine-arginine motif) CXC chemokine subgroup (Cole et al., 1998). The induction of such chemokines usually takes place in response to any immunoinflammatory condition, including infection (Widney et al., 2005; Hofer et al., 2008; Miu et al., 2008), autoimmune diseases (Patel et al., 2001), and allograft rejection (Hancock et al., 2000). The C-X-C motif chemokine receptor 3 (CXCR3) is the main receptor for such ligands in various host immune cells including activated T cells, memory T cells, and natural killer (NK) cells (Loetscher et al., 1996; Yamamoto et al., 2000; Loetscher et al., 2001). In addition to stimulation of activated T cells and NK cells, CXCR3 ligands have a variety of non-chemotactic activities, such as inhibition of angiogenesis (Strieter et al., 1995), stimulation of T lymphocyte proliferation and interferon γ (IFN-γ) production by leukocytes (Whiting et al., 2004), and antimicrobial activity (Cole et al., 2001). These chemokines are induced by a wide spectrum of neuroimmune diseases of the central nervous system (CNS) in humans and related animal models (Lahrtz et al., 1997; Amin et al., 2009). Previous studies reported expression of CXCL9 in microglia and CXCL10 in astrocytes (Hofer et al., 2008; Christensen et al., 2009), while other studies reported the existence of CXCR3 receptors on microglia (Rappert et al., 2004; de Jong et al., 2008) and likely nerve cells (Nelson and Gruol, 2004). The majority of CD8^+^ T cells in cerebrospinal fluid express CXCR3 (de Lemos et al., 2005; Christensen et al., 2006). Taken together, CXCR3 and its ligands promote T cell trafficking in cell-mediated immunity, making it reasonable to believe that these chemokines may have a vital role in the pathogenesis of parasitic infections in the CNS (Rosas et al., 2005; Amin et al., 2008; Oghumu et al., 2015).

Several previous studies have investigated the role of the CXCR3 for a variety of different infectious diseases (Widney et al., 2005; Hofer et al., 2008; Miu et al., 2008). In parasitic infection including some important members of apicomplexan parasites, the expression of CXCR3 was highly up-regulated on CD4^+^ and CD8^+^ T cells in the spleen during *Plasmodium berghei-*mediated cerebral malaria (Hansen et al., 2007). Also, an obvious role of the CXCR3 in controlling parasite replication during acute *P. chabaudi* AS infection has been reported (Berretta et al., 2019). In cases of infection with *Toxoplasma gondii*, which is closely related to the intracellular protozoan parasite *N. caninum*, CXCR3 deficiency results in the loss of ability to control intestinal infection (Cohen et al., 2013), increased brain parasite burden (Umeda et al., 2021), and severe embryonic damage in a pregnant model (Nishida et al., 2021).

CXCR3 and its ligands play a fundamental role in many pathological conditions of the CNS involving inflammation (Muller et al., 2010). Many cytokines, including IFN-γ, may regulate expression of the CXCR3 receptor and its ligands (Luster et al., 1985; Farber, 1990; Nakajima et al., 2002) and the protective immunity against *N. caninum* involves the Th1-type immune response. To date, based on our knowledge, no report has described the possible role of CXCR3 against *N. caninum* infection. In the current study, we investigate this point using C57BL/6 mice and CXCR3-knockout (CXCR3KO) mice.

## Materials and methods

### Ethics statement

**Figure 1:**
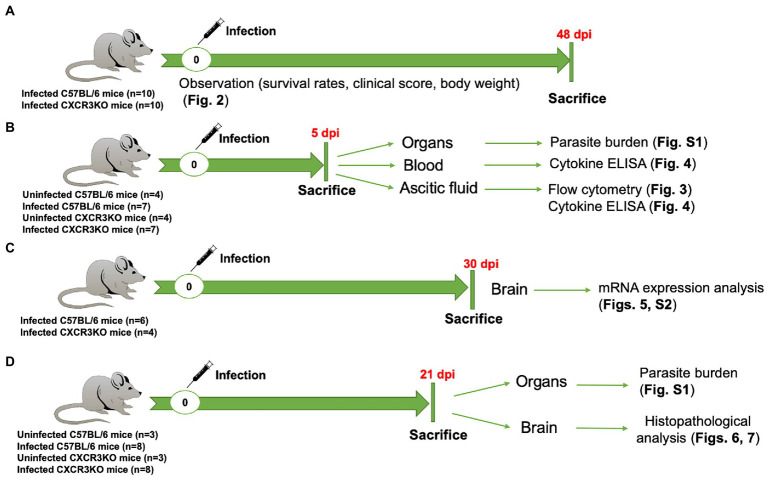
Entire experimental design in this study. **(A)** Survival experiment, 10 C57BL/6 mice (WT) and 10 CXCR3KO mice (KO) were intraperitoneally (i.p) infected with *Neospora caninum* tachyzoites (1 × 10^6^). Mice were monitored daily for survival rate, body weight, and clinical scores. All mice were sacrificed at 48 days post infection (dpi). **(B)** WT (*n* = 7) and KO mice (*n* = 7) were i.p infected with *N. caninum* tachyzoites (1 × 10^6^). Four uninfected mice from each strain were used as control groups. All mice were sacrificed at 5 dpi. Ascitic fluid, blood and different organs (brain, spleen, lung, and liver) were collected for examination of parasite burden, flow cytometry and cytokine ELISAs. **(C)** mRNA expression analysis of selective host genes in brain samples. Although six WT mice and six KO mice were infected with *N. caninum*, euthanasia was performed due to human endpoints for two CXCR3KO mice before sampling. Therefore, *N. caninum*-infected WT (*n* = 6) and KO mice (*n* = 4) were sacrificed at 30 dpi. Brains of infected mice were collected and subjected to RNA extraction. **(D)** Eight WT mice and eight KO mice were infected with 1 × 10^6^ *N. caninum* tachyzoites and sacrificed at 21 dpi. Three uninfected WT mice and three uninfected KO mice were used as control groups. Different organs (brain, spleen, lung, and liver) were collected for examination of parasite burden (right half) and histopathological analysis (left half of brain).

The study was performed in accordance with recommendations of the Guide for the Care and Use of Laboratory Animals of the Ministry of Education, Culture, Sports, Science and Technology, Japan. The protocol was approved by the Committee on the Ethics of Animal Experiments at Obihiro University of Agriculture and Veterinary Medicine, Hokkaido, Japan (Permit No. 21-38, 20-24, 19-53, 18-42, 29-44). For mice, all experiments were performed using general anesthesia with isoflurane prior to challenge with the *N. caninum* parasite and cervical dislocation as a common method of animal euthanasia. For human endpoints, euthanasia by central destruction is performed before the animal becomes unconscious or unresponsive to external stimuli, when it loses more than 20% of its body weight, or when it has severe difficulty walking. Entire experimental design and the related data were shown in Figure 1 and Supplementary Data Set, respectively.

### Parasites and cell cultures

*Neospora caninum* tachyzoites (Nc-1 strain, the passage number is less than 40) were propagated in monolayers of Vero cells cultured in Eagle’s Minimum Essential Medium (Sigma-Aldrich, St. Louis, MO, United States) with 8% heat-inactivated fetal bovine serum (Biowest, Nuaille, France) and 1% penicillin–streptomycin (Sigma-Aldrich). For the purification of tachyzoites, intracellular parasites and host cell debris were harvested after washing with cold sterile phosphate-buffered saline (PBS), and the final pellet was resuspended in Roswell Park Memorial Institute 1640 (RPMI-1640) medium (Sigma-Aldrich), passed through a 27-gauge, and then filtered using a 5.0-μm pore-size filter (Millipore, Bedford, MA, United States).

### Animals

C57BL/6 J mice were purchased from Clea Japan (Tokyo, Japan). CXCR3KO mice backcrossed to C57BL/6 were purchased from Jackson Laboratory (Bar Harbor, ME, USA). All animals were housed in cages under specific pathogen-free conditions in the animal facility of the National Research Center for Protozoan Diseases at Obihiro University of Agriculture and Veterinary Medicine. Female mice (9–11 weeks old) were used for *in vivo* experiments because of its susceptibility to *N. caninum* infection. For mouse experimental samples, mice were divided into four infection stages as uninfected, acute stage (5 dpi), subacute stage (21–30 dpi) and chronic stage (48 dpi; Fereig et al., 2021).

### Mice infection with *Neospora caninum*

For mouse survival experiment (Figure 1A), female C57BL/6 wild-type (WT) mice and female CXCR3KO mice (Total number = 10 mice per group from two independent trials, 5 animals/group/trial) were injected intraperitoneally with 1 × 10^6^ *N. caninum* tachyzoites suspended in 400 μl of RPMI-1640 medium. Survival, body weight, and clinical scores of all infected mice were monitored daily for 48 days. Clinical scores were assigned based on hunching, piloerection, dispersed and shaggy appearance, warm-seeking behavior, ptosis, sunken eyes, ataxia, latency of movement, flaccidity, touch reflexes, latent skin and eye reflexes, and mice lying on their belly. Scores varied from 0 (no signs) to 10 (all signs; Hermes et al., 2008). Finally, cervical dislocation was used to sacrifice surviving mice.

For examination of parasite burden, flow cytometry and cytokine ELISAs (Figure 1B), female WT mice and female CXCR3KO mice (4 mice and 3 mice per group from two independent trials, total 7 mice/group) were injected intraperitoneally with 1 × 10^6^ *N. caninum* tachyzoites suspended in 400 μl of RPMI-1640 medium. Four uninfected mice from each strain were used as control groups. All mice were sacrificed at 5 dpi. Ascitic fluid, blood and different organs (brain, spleen, lung, and liver) were collected.

For mRNA expression analysis of selective host genes in brain samples (Figure 1C), female WT mice and female CXCR3KO mice (6 mice per group from one trial) were injected intraperitoneally with 1 × 10^6^ *N. caninum* tachyzoites suspended in 400 μl of RPMI-1640 medium. Because euthanasia was performed due to human endpoints for two CXCR3KO mice, brain tissues of *N. caninum*-infected WT mice (*n* = 6) and CXCR3KO mice (*n* = 4) was collected at 30 dpi and subjected to RNA extraction.

For examination of parasite burden and histopathological analysis (Figure 1D), female WT mice and female CXCR3KO mice (8 mice per group from one trial) were injected intraperitoneally with 1 × 10^6^ *N. caninum* tachyzoites suspended in 400 μl of RPMI-1640 medium. Three uninfected mice from each strain were used as control groups. Different organs (brain, spleen, lung and liver) were collected for examination of parasite burden (right half) and histopathological analysis (left half of brain).

### Flow cytometric analysis

The mice were euthanized and peritoneal cells of infected mice were harvested in 5 ml of ice-cold PBS. The cells were blocked with anti-mouse CD16/CD32 (FcBlock™, clone 2.4G2) to avoid non-specific binding of monoclonal antibodies (mAbs) to Fc receptors, and then incubated with the following mAbs: phycoerythrin (PE)-conjugated anti-mouse CD11b (clone M1/70), PE-conjugated anti-mouse CD11c (clone HL3), PE-conjugated anti-mouse NK1.1 (clone PK136), PE-conjugated anti-mouse CD8a (clone 58–6.7), fluorescein isothiocyanate (FITC) or PE-conjugated anti-mouse CD3e (clone 145-2C11), and FITC-conjugated anti-mouse CD4 (clone RM4-5) for 30 min at 4°C. Stained cells (monocyte/macrophage/type 2 conventional dendritic cells, CD11b^+^; dendritic cell, CD11c^+^; NK cell, CD3^−^ NK1.1^+^; natural killer T (NKT) cell, CD3^+^ NK1.1^+^; CD4^+^ T cell, CD3^+^ CD4^+^; and CD8^+^ T cell, CD3^+^ CD8^+^) were washed with cold PBS, fixed with 0.5% paraformaldehyde in PBS, and examined with an EPICS XL flow cytometer (Beckman Coulter, Pasadena, CA, United States). Absolute numbers of cells expressing each marker were calculated. All antibodies were purchased from BD Pharmingen (San Diego, CA, United States).

### Cytokine ELISA

Concentrations of interleukin-6 (IL-6) and IFN-γ in ascites and serum samples collected at 5 dpi were measured using commercial cytokine enzyme-linked immunosorbent assay (ELISA) kits (Mouse OptEIA ELISA set; BD Biosciences, San Jose, CA, United States) according to the manufacturer’s recommendations. Cytokine standards were analyzed on the same plates to generate a curve for calculations of cytokine concentrations.

### DNA isolation and quantitative real-time PCR

The mice were euthanized, and their brains, spleens, lungs and livers were collected. Organs from uninfected mice were used as negative controls and included during DNA extraction procedures and qPCR. Total genomic DNA was extracted from all collected tissue as previously described (Nishikawa et al., 2018), with slight modifications. Approximately 5 ml of lysis buffer [0.1 M Tris–HCl (pH 9.0), 1% sodium dodecyl sulfate, 0.1 M NaCl, 1 mM EDTA] and 100 μg/ml proteinase K were added to tubes containing the whole organ or fragment (0.5 g). The mixture was incubated at 55°C for a few days. Thereafter, DNA was purified by phenol:chloroform:isoamyl alcohol (Nakarai Tesque, Kyoto, Japan) and ethanol precipitation. Concentrations of DNA obtained from tissues were determined with a spectrophotometer. To quantify parasite burdens in collected tissues, we used primers for *Nc5* previously described (Collantes-Fernández et al., 2002; Supplementary Table S1) to carry out PCR in a final volume of 10 μl containing 0.2 μl (final 500 nM) of forward and reverse primers, 4.8 μl of 1 × SYBER GREEN reaction master mix (Applied Biosystems, Waltham, MA), and 5 μl (50 ng) of template DNA. A standard curve constructed from 10-fold serial dilutions of *N. caninum* DNA extracted from 1 × 10^5^ parasites was used to calculate the number of parasites per 50 ng of tissue DNA.

### Histopathological and immune histopathological analysis of mouse brain

Brains were removed from the sacrificed mice, fixed in 10% phosphate-buffered formaldehyde, and embedded in paraffin. Paraffin-embedded tissue sections (3 μm thick) were prepared from the frontal lobes, striatum, and diencephalon, and then stained with hematoxylin and eosin (H&E). Percentages of inflammatory cell infiltration, necrosis, and meningitis were examined in all groups.

Immunohistochemical staining was used to quantify populations of macrophages, astrocytes, and microglia using rabbit polyclonal antibodies against CD3, glial fibrillary acidic protein (GFAP; Thermo Fisher Scientific, Waltham, MA, United States), and ionized calcium-binding adapter molecule 1 (Iba1; Fujifilm Wako Pure Chemical Corporation, Richmond, VA, United States), respectively, using ImmunoSaver antigen activation (Nisshin EM, Tokyo, Japan). For detection of regulatory T (Treg) cells, immunohistochemical staining was performed using a rat monoclonal antibody against forkhead box P3 (FoxP3; clone FJK-16 s, Thermo Fisher Scientific) with antigen retrieval at 98°C for 15 min in citrate buffer (pH 6.0) in a microwave oven, followed by counterstaining with Mayer’s hematoxylin. For quantification analysis, 10 images of the cerebral cortex of each mouse were randomly taken at 400× magnification. Positive areas were quantified using Adobe Photoshop (Adobe Systems, San Jose, CA, United States) and ImageJ.^1^ To detect proportions of Treg cells relative to all infiltrating cells, rates of nuclear positivity for FoxP3 relative to Mayer’s hematoxylin were quantified using Adobe Photoshop and ImageJ.

### Real-time reverse transcription–polymerase chain reaction

RNA from brain tissues was extracted by TRIZOL (Sigma-Aldrich) according to the manufacturer’s instructions. The purity and concentration of RNA were quantified using a NanoDrop (Thermo Fisher Scientific). A Superscript II Reverse Transcriptase kit (Invitrogen, Carlsbad, CA, United States) was used for cDNA synthesis according to the manufacturer’s protocol. Primer sequences were designed by Primer Express Software (Applied Biosystems, Waltham, MA, United States; Supplementary Table S1). Real-time polymerase chain reaction (PCR) was performed using SYBR™ Green super mix (Applied Biosystems). In brief, 0.1 μl of each primer, 2 μl (20 ng) of cDNA, and 5 μl of SYBR Green mix were used in a 10-μL reaction volume. Amplifications were performed using an ABI 7700 Prism Sequence Detector (Bio-Rad, Hercules, CA, United States). The cycle threshold (Ct) values were normalized to the expression levels using the 2^−∆∆Ct^ method (Kobayashi et al., 2019; Umeda et al., 2021). Glyceraldehyde-3-phosphate dehydrogenase (*Gapdh*) was used as the internal control gene after comparison with *Actb* using RefFinder (Xie et al., 2012). Normalized mRNA expression values were obtained by subtracting the mRNA expression level of *Gapdh* from that of the target gene. The calibrator sample was the infected WT group.

### Statistical analysis

Statistical analysis was performed using Prism 5.0 software (GraphPad Software, La Jolla, CA, United States). A log-rank test was used for group comparisons of survival rates. Results of flow cytometry and cytokine ELISA were analyzed with two-way ANOVA followed by Tukey–Kramer multiple-comparison test. Areas positive for different cell markers in brain tissues were analyzed with two-way ANOVA plus Bonferroni’s multiple comparison test. mRNA analysis results were compared by two-tailed unpaired Student’s t-test or Mann–Whitney *U*-test. Levels of statistical significance (*p*-value < 0.05) are presented with asterisks defined in each Figure legend.

## Results

### High mortality rate of CXCR3KO mice infected with *Neospora caninum*

**Figure 2:**
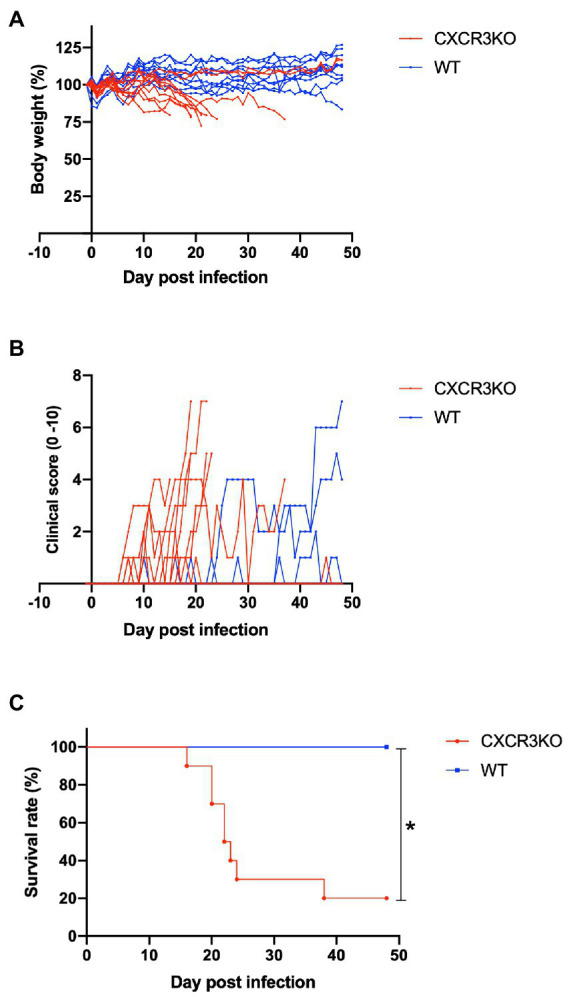
Clinical parameters of mice infected with *Neospora caninum*. Wild-type (WT; *n* = 10) and CXCR3-knockout (CXCR3KO) mice (*n* = 10) were intraperitoneally infected with *N. caninum* tachyzoites. Alterations of body weight **(A)**, clinical scores **(B)**, and survival rate **(C)** of infected mice for 50 days post infection (dpi) were measured. *Significant differences (*p* < 0.05) between groups were analyzed with log-rank test **(C)**.

To assess roles of the chemokine receptor CXCR3 in the pathogenesis of *Neospora caninum* infection, infected mice were monitored daily for survival, body weight, and clinical scores for 48 days (Figure 2). Knockout of the CXCR3 gene deteriorated the health condition of infected mice, as reflected by low body weights and early observation of clinical symptoms (Figures 2A,B). Moreover, 80% of CXCR3KO mice succumbed to the infection, while 100% of WT mice survived until the end of the experiment (*p* < 0.05, Figure 2C). This result indicates CXCR3KO mice as having high susceptibility of *N. caninum* infection compared with WT mice.

### Cell recruitment in peritoneal exudates of mice during early stage of infection

**Figure 3:**
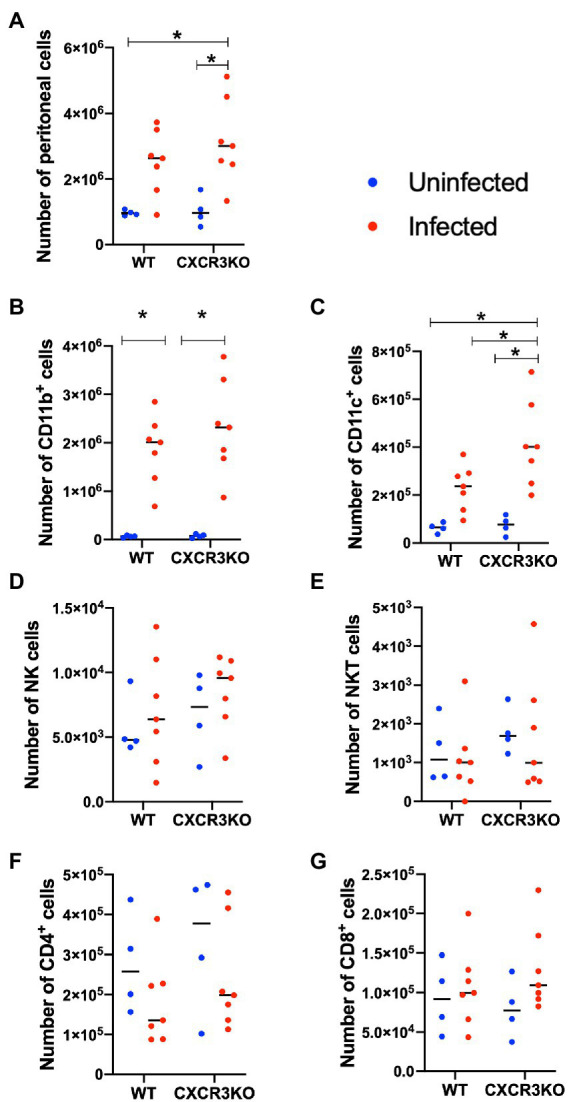
Recruitment of immune cells in ascitic fluid of mice following *Neospora caninum* infection. Wild-type (WT) and CXCR3-knockout (CXCR3KO) mice were infected intraperitoneally with *N. caninum* tachyzoites. After 5 days, peritoneal exudate cells of infected mice (*n* = 7) and uninfected mice (*n* = 4) were collected. Cells were analyzed by flow cytometry to determine absolute numbers of peritoneal cells **(A)**, CD11b^+^ cells **(B)**, CD11c^+^ cells **(C)**, NK cells **(D)**, NKT cells **(E)**, CD4^+^ cells **(F)**, and CD8^+^ cells **(G)**. Values per individual (symbols) and mean levels (horizontal lines) are shown. Data were analyzed with two-way ANOVA followed by Tukey–Kramer multiple-comparison test. *p < 0.05.

Here, we investigated the role of CXCR3 in recruitment of leukocytes and T cells during the early stage of infection (5 dpi; Figure 3). Infection with *N. caninum* increased numbers of peritoneal cells, especially in CXCR3KO mice (*p* < 0.05, Figure 3A). Numbers of CD11b^+^ cells were significantly increased after infection in both strains of mice (*p* < 0.05, Figure 3B). However, the peritoneal cavity of infected CXCR3KO mice displayed increased numbers of CD11c^+^ cells (dendritic cells) compared with that of infected WT mice (*p* < 0.05, Figure 3C). Absolute numbers of NK cells (Figure 3D), NKT cells (Figure 3E), CD4^+^ cells (Figure 3F), and CD8^+^ cells (Figure 3G) did not differ between WT and CXCR3KO mice.

### Concentrations of IL-6 and IFN-γ in ascites and sera during early stage of infection

**Figure 4:**
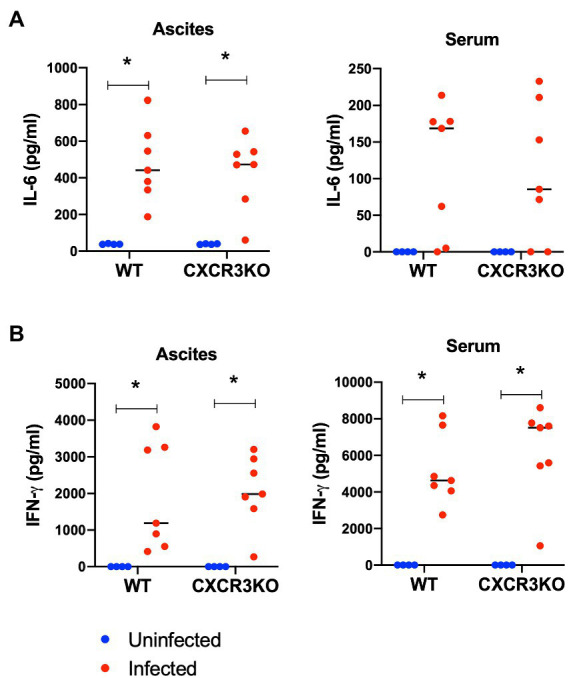
Concentrations of IL-6 and IFN-γ in sera and ascites of mice following *Neospora caninum* infection. Wild-type (WT) and CXCR3-knockout (CXCR3KO) mice were infected intraperitoneally with *N. caninum* tachyzoites. After 5 days, the sera and ascites of infected mice (*n* = 7) and uninfected mice (*n* = 4) were collected. Concentrations of IL-6 **(A)** and IFN-γ **(B)** were measured. Values per individual (symbols) and mean levels (horizontal lines) are shown. Data were analyzed with two-way ANOVA followed by Tukey–Kramer multiple-comparison test. *p < 0.05.

To examine the effect of CXCR3 deficiency on inflammatory cytokine production, ascites fluids and sera of mice were collected at 5 dpi (acute stage) and examined for inflammatory markers IL-6 and IFN-γ using ELISA (Figure 4). Infection with *N. caninum* resulted in production of IL-6 in ascites and IFN-γ in ascites and sera at 5 dpi in both mouse strains (*p* < 0.05). However, no significant difference in concentrations of these cytokines was observed between WT and CXCR3KO mice.

### Parasite load in different organs

To examine the effects of CXCR3 on the parasite load within mouse survival time, the parasite numbers in brain, spleen, lungs, and liver tissues of infected mice were quantified at 5 dpi (acute stage) and 21 dpi (subacute stage) by quantitative PCR analysis (Supplementary Figure S1). However, no significant difference in parasite loads of test samples was observed.

### Gene expression analysis of selective host genes in brain

**Figure 5:**
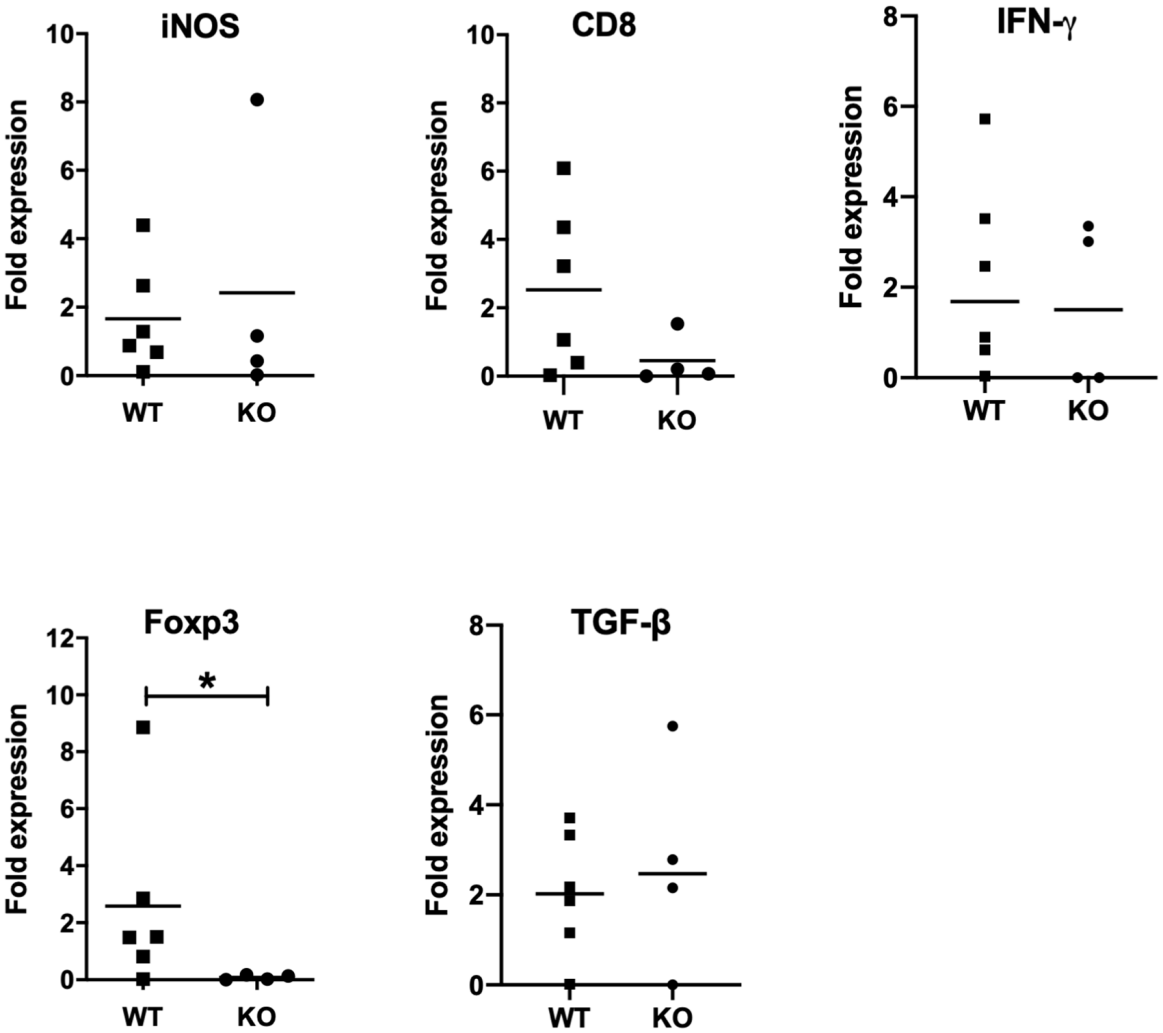
mRNA expression analysis of selective host genes in brain samples. Brain samples were collected from *Neospora caninum*-infected wild-type (WT) and CXCR3-knockout (KO) mice 30 days after the infection. All collected samples were subjected to mRNA expression analysis using specific primers for genes related to brain pathology. Expression levels of marker genes related to brain pathology were compared. Fold expression per individual (symbols) and mean levels (horizontal lines) against WT mice are shown (WT, *n* = 6, KO, *n* = 4). Expression of IL-10 mRNA was not detected. Data were analyzed with the Mann–Whitney *U*-test or *t*-test. *p < 0.05.

Due to the period of mouse dying from 25 to 29 dpi (Figure 2C), mRNA expression of key host genes in the brains of mice was examined at 30 dpi (subacute stage) by real-time PCR to determine the precise function of CXCR3 in *N. caninum* infection (Supplementary Figure S2; Figure 5). Our previous transcriptome analysis reported that genes encoding chemokines and chemokine receptors (*Ccl8*, *Ccl5*, *Cxcl10*, and *Cxcr6*), IFN-inducible GTPase family members (*Tgtp2*, *Gbp8*, and *Iigp1*), and serum amyloid A3 (*Saa3*) were significantly upregulated in the brain by *N. caninum* infection (Nishimura et al., 2015). However, there was no significant difference in expression levels between infected WT mice and infected CXCR3KO mice (Supplementary Figure S2A). Solute carrier family members 4 and 5 (*Slc6a4* and *Slc6a5*), tryptophan hydroxylase 2 (*Tph2*), and low-density lipoprotein receptor (*Ldlr*) were significantly altered in brain samples from mice exhibiting clinical signs of neosporosis (Nishimura et al., 2015). However, no difference in expression levels was observed between infected CXCR3KO mice and infected WT mice in the present study (Supplementary Figure S2B).

Next, we measured expression levels of marker genes related to brain pathology such as IFN-γ, inducible nitric oxide synthase (iNOS), CD8 and *FoxP3* (Figure 5). Although expression levels of iNOS, CD8 and IFN-γ showed no significant difference between infected CXCR3KO mice and infected WT mice, *FoxP3* expression was significantly decreased in infected CXCR3KO mice compared with that in infected WT mice (*p* < 0.05). Although FoxP3^+^ Treg cells secrete some inhibitory factors including transforming growth factor-β (TGF-β) and IL-10 (Arranz-Solís et al., 2021; Gao et al., 2021), there was no significant difference in the mRNA expression of TGF-β (Figure 4) and expression of IL-10 was not detected.

### Pathological analysis of brain tissue

Pathological analysis of brains was performed at 21 dpi (subacute stage). H&E staining revealed focal necrosis and non-suppurative inflammation were induced by *N. caninum* infection in brain tissues of both mice (Supplementary Table S2). Inflammatory cell infiltration, necrosis and meningitis were observed in brain tissues of both mouse strains infected with *N. caninum*. Moreover, the number of the infected CXCR3KO mice exhibiting necrosis lesions was higher compared with the infected WT mice (*p* < 0.05).

**Figure 6:**
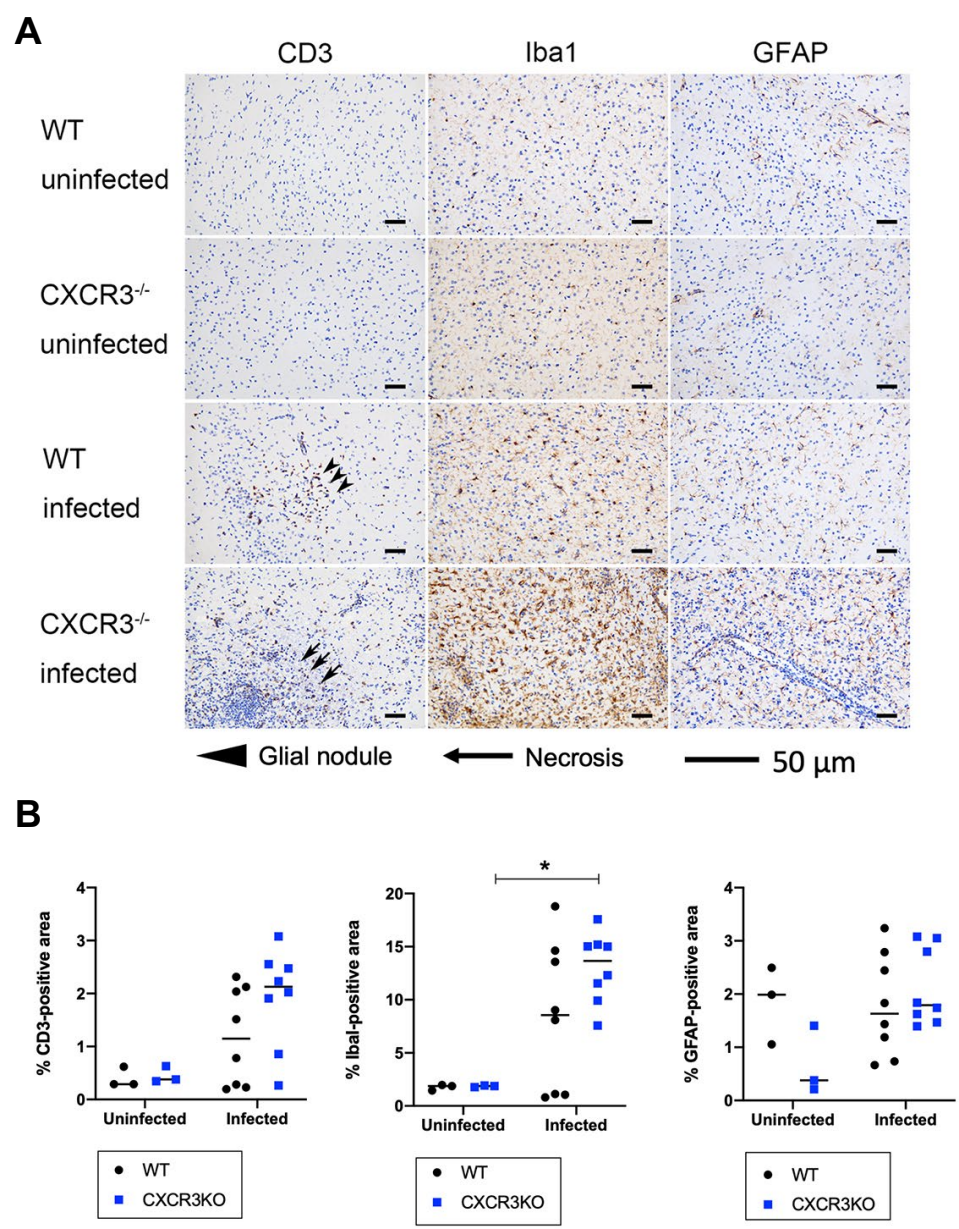
T cell, astrocyte, and microglia populations in brain (frontal lobes, striatum, and diencephalon) of mice. Brains from wild-type (WT) and CXCR3-knockout (CXCR3KO) mice surviving at 21 days after infection (*n* = 8), as well as brains from uninfected mice (*n* = 3), were subjected to histopathological processing. T cell, astrocyte, and microglia populations were identified using immunohistochemical staining for CD3, ionized calcium-binding adapter molecule 1 (Iba1), and glial fibrillary acidic protein (GFAP), respectively. **(A)** Representative immunohistochemical images were taken from serial tissue sections containing T cells, astrocytes, and microglia. Glial nodules and necrosis are shown as arrowheads and arrows, respectively. Scale bars, 50 μm. **(B)** Positive areas were compared between WT and CXCR3KO or infected and uninfected mice. Horizontal bars represent the mean value. Data were analyzed with two-way ANOVA followed by the Tukey–Kramer multiple-comparison test. *p < 0.05.

Populations of T cells (CD3^+^), microglia (Iba1^+^), and astrocytes (GFAP^+^) were analyzed by immunohistochemistry (Figure 6). The results indicated no significant differences in positive areas between CXCR3KO and WT mice for all examined cell types. However, significantly more microglia were observed in infected CXCR3KO mice than in uninfected CXCR3KO mice (*p* < 0.05, Figure 6B).

**Figure 7:**
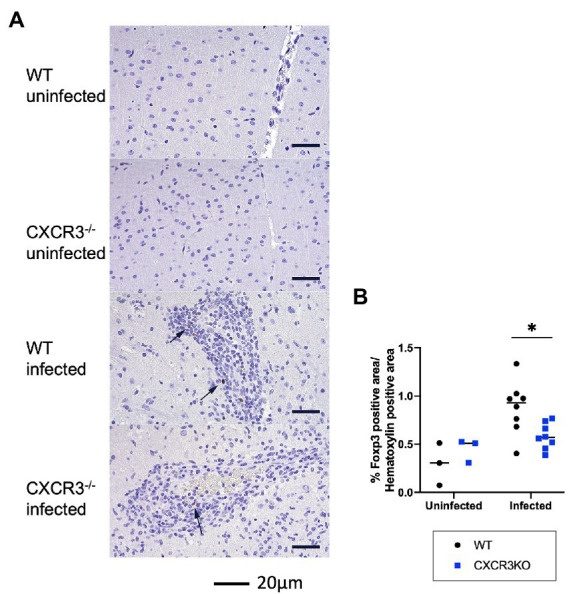
Population of FoxP3^+^ cells in mouse brains. **(A)** Brain regulatory T cells were identified by immunohistochemistry for FoxP3 and counterstaining with Mayer’s hematoxylin. Nuclear positivity for FoxP3 was detected in the brains of infected wild-type (WT) and CXCR3-knockout (CXCR3KO) mice (arrowheads). **(B)** To clarify the proportion of Treg cells to other infiltrating cells, proportions of nuclear-positive area for FoxP3 to hematoxylin were analyzed with two-way ANOVA followed by the Tukey–Kramer multiple-comparison test. *p < 0.05.

As shown in Figure 5, induction of Treg cells (FoxP3^+^) may be inhibited in infected CXCR3KO mice. Immunohistochemistry showed that the proportion of FoxP3^+^ area to Mayer’s hematoxylin-positive area was significantly decreased in infected CXCR3KO mice compared with that in infected WT mice (*p* < 0.05, Figure 7).

## Discussion

The absence of CXCR3 gene potentially affects the recruitment of immune cells to sites of *N. caninum* infection in mice. Our flow cytometric analysis of peritoneal cells of infected mice at 5 dpi showed no significant differences in recruitment of CD11b^+^ cells between WT and CXCR3KO mice. However, the peritoneal fluid of CXCR3KO mice displayed a significant increase in the population of CD11c^+^ cells compared with that of WT mice at 5 dpi. Because a previous study showed that infected dendritic cells facilitate systemic dissemination of *N. caninum* in mice (Collantes-Fernandez et al., 2012), we suggest a high degree of parasite infection occurs *via* infected dendritic cells in infected-CXCR3KO mice. However, an increase of peritoneal CD11c^+^ cells may not contribute on the pathogenesis during the acute stage of *N. caninum* infection in this study.

Several studies have reported a protective role of Th1-type immunity against *N. caninum* infection, as represented by a high production of inflammatory cytokines (Baszler et al., 1999; Nishikawa, 2017). However, no differences in levels of IFN-γ or IL-6 in ascites fluids and serum samples of WT and CXCR3KO mice were identified at 5 dpi. This result indicates few roles for CXCR3 in the innate immunity of mice against *N. caninum* infection. Moreover, no significant differences in parasite load were detected for brain, spleen, lungs, or liver tissues of either mouse strain at 5 dpi or 21 dpi, suggesting no correlation between the parasite burden and mouse survival in CXCR3KO mice.

*Neospora caninum* infection induces a T-cell immune response in infected tissues that is mediated by IL-12 and IFN-γ (Khan et al., 1997; Nishikawa et al., 2001). CD8^+^ T cells control intracellular parasite growth in various organs (Correia et al., 2015). In the brain of an infected host, *N. caninum* encourages abnormal production of glial cells (astrocytes and microglia) with high production of IL-10, tumor necrosis factor α, and nitric oxide (NO; Pinheiro et al., 2006, 2010). Stimulation of glial cells with IFN-γ controls growth of the parasite independent of NO production (Jesus et al., 2013). Despite this, NO can facilitate diffusion of the parasite by inducing changes in blood–brain barrier permeability (Lagrange et al., 1999; De Jesus et al., 2014). In our study, the brain tissues of infected CXCR3KO mice displayed increased populations of microglia compared with those of non-infected CXCR3KO mice. This result may reflect the severity of infection in CXCR3KO mice. Nevertheless, non-significant results were observed between WT and CXCR3KO mice.

The results of our histopathological analysis provide some evidence regarding the protective mechanism of CXCR3 in C57BL/6 mice. More necrosis was observed in infected CXCR3KO mice than infected WT mice at the subacute stage of infection (21 dpi). Moreover, the brain tissues of infected CXCR3KO mice displayed an increased tendency for cell infiltration compared with those of infected WT mice. Taken together, direct brain tissue damage is the main suggested cause of the higher mortality rate in infected CXCR3KO mice compared with that in WT mice.

In viral infections, such as lymphocytic choriomeningitis virus and West Nile virus (WNV) encephalitis, CXCR3 deficiency can affect numbers of CNS-infiltrating CD4^+^ and CD8^+^ T cells. The reduction in migration of CXCR3-expressing CD8^+^ T cells into the cerebellum was the main cause for CXCR3KO mice displaying increased mortality rates following infection with WNV (Christensen et al., 2004; Zhang et al., 2008). In another study, the authors demonstrated a protective role for CXCR3 in experimental autoimmune encephalomyelitis (EAE) using C57BL/6 mice (Muller et al., 2007). CXCR3KO mice had more severe demyelination and axonal damage with high diffused inflammatory lesions in the CNS compared with WT mice. The same study detected significantly lower levels of FoxP3^+^ Treg cells in the cerebellum and spinal cord of CXCR3KO mice with EAE, notably, without differences of IFN-γ levels in CNS. The authors suggested that CXCR3 is required for Treg cell recruitment in the CNS, whereby it facilitates the interaction of these cells with effector T cells. The loss of CXCR3 signaling in CXCR3KO mice increased autoimmune-mediated tissue damage by limiting Treg cell spread. Given the evidence discussed above, it is reasonable to believe that activated T cells in infected brains of WT mice are CXCR3^+^ Treg cells.

FoxP3^+^ Treg cells primarily modulate the immune response by secreting inhibitory factors (e.g., TGF-β, IL-10, and amphiregulin) or inhibiting inflammatory cytokines produced by Th1/Th17 cells (e.g., IFN-γ, IL-17, and IL-23) to protect against their harmful effects (Ito et al., 2019; Gao et al., 2021). In the current study, a significant reduction in FoxP3 expression levels was detected in the brain tissues of infected CXCR3KO mice at 30 dpi. In addition, necrosis was more frequently observed in infected CXCR3KO mice, indicating that the downregulation of Treg cells promoted excessive inflammation and resulted in necrosis. However, significant difference in mRNA expression of TGF-β and IL-10 was not seen in our study. Therefore, further examination of Treg cells in the brain following *N. caninum* infection is needed.

Previous studies have mentioned the relation between the raise of CD11c^+^ cells and low recruitment of Treg cells in CNS using closely related parasite, *Toxoplasma gondii* (O'Brien et al., 2017). The treatment of mice with integrin LFA-1 blocking antibodies, which reduced the number of CD11c^+^ cells consist of a mixture of microglia and dendritic cells (John et al., 2011), resulted in a significant increase in the speed of FoxP3^+^ cell migration in the brain of infected mice. These results suggest that interactions between Treg cells and CD11c^+^ cells limit the migratory speed of Treg cells in the CNS (O'Brien et al., 2017). Thus, the similar action can be expected here in *Neospora* infection while further examination of the interaction between CD11c^+^ cells and Treg cells in CNS is needed.

## Conclusion

In *N. caninum*-infected wild-type mice, brain Treg cells might promote neurological recovery during the chronic stage of infection and might reduce necrosis in brain tissue compared with the infected CXCR3KO mice. Therefore, CXCR3 play an important role for Treg cells to decrease brain damage following *N. caninum* infection. However, more studies are required to elucidate the detailed mechanism of such protective efficacy.

## Data availability statement

The original contributions presented in the study are included in the article/Supplementary material, further inquiries can be directed to the corresponding author.

## Ethics statement

The animal study was reviewed and approved by Committee on the Ethics of Animal Experiments at Obihiro University of Agriculture and Veterinary Medicine, Hokkaido, Japan.

## Author contributions

HA was involved in data curation, formal analysis, investigation, and writing—original draft. SM, MM, and HF contributed to data curation and investigation. KW and NU contributed to data curation, investigation, and writing—review and editing. YN was involved in data curation, formal analysis, investigation, writing—review and editing, conceptualization, project administration, funding acquisition, and supervision. All authors contributed to the article and approved the submitted version.

## Funding

This study was supported in part by the Research Program on Emerging and Re-emerging Infectious Diseases [21fk0108137h (YN)] from the Agency for Medical Research and Development (AMED), and KAKENHI Grants from the Japan Society for the Promotion of Science [20K21359 and 21H02353 (YN)].

## Conflict of interest

The authors declare that the research was conducted in the absence of any commercial or financial relationships that could be construed as a potential conflict of interest.

## Publisher’s note

All claims expressed in this article are solely those of the authors and do not necessarily represent those of their affiliated organizations, or those of the publisher, the editors and the reviewers. Any product that may be evaluated in this article, or claim that may be made by its manufacturer, is not guaranteed or endorsed by the publisher.

## Footnotes

^1^ http://imagej.nih.gov
