# Supplementary material for: Involvement of chemokine receptor CXCR3 in the defense mechanism against Neospora caninum infection in C57BL/6 mice
Source: Front Microbiol. 2023 Jan 10;13:1045106. doi: 10.3389/fmicb.2022.1045106 (PMC9873264; doi:10.3389/fmicb.2022.1045106)
Supplement: Supplementary file 1 [file Data_Sheet_1.DOCX]

**Supplemental information file**

**Materials and methods**

**DNA extraction and quantitative PCR analysis of *N. caninum* distribution**

DNA was extracted from the tissues (brain, spleen, lungs and liver tissues) as follows. Each tissue or organ was thawed in 10 volumes of extraction buffer (0.1 M Tris-HCl [pH 9.0], 1% SDS, 0.1 M NaCl, 1 mM EDTA) and 100 μg/ml proteinase K at 55°C. The DNA was purified with phenol-chloroform extraction and ethanol precipitation. The parasite DNA was then amplified with primers specific to the *N. caninum* Nc5 gene (Table S1). Amplification, data acquisition, and data analysis were performed in the ABI Prism 7900HT sequence detection system (Applied Biosystems), and the cycle threshold values (CT) were calculated as described previously (Nishimura et al., 2015). The parasite number was calculated from the standard curve based on each CT values ranged from 0.01 to 10,000 parasites. Results of parasite loads in different tissues were analyzed with the Mann–Whitney U test or t test.

**Table S1: Sequences of primers used for this study**

| **Target gene** | **Sense primer (5'-3')** | **Anti-sense primer (5'-3')** |
| --- | --- | --- |
| *Gapdh* | TGT GTC CGT CGT GGA TCT GA | CCT GCT TCA CCA CCT TCT TGA T |
| *Ccl8* | CTG GGC CAG ATA AGG CTC C | CAT GGG GCA CTG GAT ATT GT |
| *Ccl5* | CCA ATC TTG CAG TCG TGT TTG T | CAT CTC CAA ATA GTT GAT GTA TTC TTG AAC |
| *Cxcl10* | GAC GGT CCG CTG CAA CTG | CTT CCC TAT GGC CCT CAT TCT |
| *Cxcr6* | CCC TGT ACT TTA TGC CTT TG | CTT GGA ACT GTC CTC AGA AG |
| *Saa3* | GCC TGG GCT GCT AAA GTC AT | TGC TCC ATG TCC CGT GAA C |
| *Tgtp2* | ACT GTC CAT CCC ACG GTC TC | GTG TCC CAG GAA GAA CTG CAC |
| *Gbp8* | TTT GAT GAA CCG TCT GGC AG | GTG GAG CCC AGA GGG AAA C |
| *Ligp1* | AGC CAA GAG CAC ACC GAG G | GTG GAT GCA CTC ACT GCT CCT |
| *Slc6a4* | CAA CTC CGG CTT TTC CAA TA | ATT TCC GTT GGT GTT TCA GG |
| *Slc6a5* | GGT TCA ATC TGT TGT CCG CA | TTT CCT TGG GAG CAC TGC A |
| *Tph2* | TGG GGA TTT GAT GCC TAG AAC C | TGG GTT CTT TAG AGC ATT TTT GTG T |
| *Ldlr* | GAC TCA TGC AGC AGG AAC GA | AGC GAT GCA TTT TCC GTC TC |
| *iNOS* | CAT TGG AAG TGA AGC GGT TCG | CAG CTG GGC TGT ACA AAC CTT |
| *CD8* | AGGA TGC TCT TGG CTC TTCC | TCA CAG GCG AAG TCC AAT |
| *Foxp3* | GAG AAA GCG GAT ACC AAA | TGT GAG GAC TAC CGAGCC |
| *IL-10* | AAG GGT TAC TTG GGT TGC | AAG GAG TTG TTT CCG TTA |
| *IFN-γ* | GCC ATC AGC AAC ATA AGC GTC | CCA CTC GGA TGA GCT CAT TGA ATG |
| *TGF-β* | TGA CGT CAC TGG AGT TGT ACG G | GGT TCA TGT CAT GGA TGG TGC |
| *Nc5* | ACT GGA GGC ACG CTG AAC AC | AAC AAT GCT TCG CAA GAG GAA |

**Table S2. Histopathological analysis of mouse brain at 21 dpi**

| Mouse group | % of inflammatory cell infiltration | % of necrosis | % of meningitis |
| --- | --- | --- | --- |
| WT, uninfected | 0 (0/3) | 0 (0/3) | 0 (0/3) |
| CXCR3^-^/^-^, uninfected | 0 (0/3) | 0 (0/3) | 0 (0/3) |
| WT, infected | 50 (4/8) | 37.5 (3/8) | 62.5 (5/8) |
| CXCR3^-^/^-^, infected | 87.5 (7/8) | 87.5 (7/8) * | 75 (6/8) |

* More necrosis was observed in infected CXCR3KO mice than WT mice by χ^2^ test (P < 0.05)

**Figure S1. Parasite burdens in tissues of wild-type (WT) and CXCR3-knockout (KO) mice at 5 days (A) and 21 days (B) post-infection with *N. caninum*.** Values indicate numbers of parasites in 50 ng of tissue DNA. Number of parasites per individual (symbols) and mean levels (horizontal lines) are shown (5 days, n = 8 for all groups; 21 days, n = 8 for all groups). Data were analyzed with the Mann–Whitney U test or t test, but no differences were detected.

**Figure S2. mRNA expression analysis of selective host genes in brain samples.** Brain samples were collected from *N. caninum*-infected wild-type (WT) and CXCR3-knockout (KO) mice 30 days after the infection. All collected samples were subjected to mRNA expression analysis using specific primers for each target gene. (A) Genes encoding chemokines and chemokine receptors upregulated in the brain by *N. caninum* infection in our previous study (Nishimura et al., 2015). (B) Genes significantly altered in brain samples from mice exhibiting clinical signs of neosporosis in our previous study (Nishimura et al., 2015). Expression levels of marker genes related to brain pathology were compared. Fold expression per individual (symbols) and mean levels (horizontal lines) against WT mice are shown (WT, n = 6, KO, n = 4). Data were analyzed with the Mann–Whitney U test or t test, but no differences were detected.
